# Competing treatments for migraine: a headache for decision-makers

**DOI:** 10.1186/s10194-023-01686-y

**Published:** 2023-12-05

**Authors:** Hema Mistry, Seyran Naghdi, Martin Underwood, Callum Duncan, Jason Madan, Manjit Matharu

**Affiliations:** 1 Warwick Clinical Trials Unit, Warwick Medical School, University of Warwick, Coventry, CV4 7AL UK; 2 University Hospitals Coventry and Warwickshire, Clifford Bridge Road, Coventry, CV2 2DX UK; 3 Department of Neurology, NHS Grampian, Aberdeen Royal Infirmary, Aberdeen, AB25 2ZN UK; 4 National Hospital for Neurology and Neurosurgery, University College London Hospitals NHS Trust, London, WC1N 3BG UK

**Keywords:** Chronic migraine, Prophylactic medications, Cost-effectiveness

## Abstract

**Background:**

Migraine is the world’s second most common disabling disorder, affecting 15% of UK adults and costing the UK over £1.5 billion per year. Several costly new drugs have been approved by National Institute for Health and Care Excellence.

**Aim:**

To assess the cost-effectiveness of drugs used to treat adults with chronic migraine.

**Methods:**

We did a systematic review of placebo-controlled trials of preventive drugs for chronic migraine. We then assessed the cost-effectiveness of the currently prescribable drugs included in the review: Onabotulinum toxin A (BTA), Eptinezumab (100mg or 300mg), Fremanezumab (monthly or quarterly dose), Galcanezumab or Topiramate, each compared to placebo, and we evaluated them jointly. We developed a Markov (state-transition) model with a three-month cycle length to estimate the costs and quality-adjusted life years (QALYs) for the different medications from a UK NHS and Personal Social Services perspective. We used a two-year time horizon with a starting age of 30 years for the patient cohort. We estimated transition probabilities based on monthly headache days using a network meta-analysis (NMA) developed by us, and from published literature. We obtained costs from published sources and applied discount rates of 3.5% to both costs and outcomes.

**Results:**

Deterministic results suggest Topiramate was the least costly option and generated slightly more QALYs than the placebo, whereas Eptinezumab 300mg was the more costly option and generated the most QALYs. After excluding dominated options, the incremental cost-effectiveness ratio (ICER) between BTA and Topiramate was £68,000 per QALY gained and the ICER between Eptinezumab 300mg and BTA was not within plausible cost-effectiveness thresholds. The cost-effectiveness acceptability frontier showed that Topiramate is the most cost-effective medication for any amount the decision maker is willing-to-pay per QALY.

**Conclusions:**

Among the various prophylactic medications for managing chronic migraine, only Topiramate was within typical cost-effectiveness threshold ranges. Further research is needed, ideally an economic evaluation alongside a randomised trial, to compare these newer, expensive CGRP MAbs with the cheaper oral medications.

**Supplementary Information:**

The online version contains supplementary material available at 10.1186/s10194-023-01686-y.

## Introduction

Migraine is one of the most common and debilitating neurological disorders globally and is the second leading cause of years lived with disability worldwide [1]. Average global migraine prevalence is reported as 12% (range: 2.6% and 21.7%), with variation between countries and between studies conducted in the same country [2]. Migraine is more common among women [3, 4] and also more prevalent among the 35–42 years age group [5]. The frequency of migraine episodes determines its classification: up to 14 migraine days per month is classified as ‘episodic’, while a headache occurring on 15 or more days per month, with at least 8 days meeting migraine criteria, is classified as ‘chronic’[6].

Chronic migraine has a disabling impact on people’s health and quality of life [7]. The global prevalence of chronic migraine is between 1.4% to 2.2% [8]. From a societal view, the more prevalent chronic migraine is, the greater the consumption of health care resources and more productivity losses. There are substantial differences in the health impact of migraine on people experiencing ≥ 15 monthly headaches days compared with people with 1–3 monthly headaches days in terms of quality of life, presenteeism of work, and total work productivity losses [5]. In the United Kingdom (UK), approximately one in six adults are affected by migraines (both episodic and chronic), predominantly young adults with personal (i.e. family) and professional responsibilities. This results in an economic burden of over £1.5 billion annually in the UK, [9] this includes both direct costs such as hospitalisation and medications, and indirect costs resulting from work presenteeism and absenteeism [10–12].

Pharmacological drugs for chronic migraine aim to reduce the frequency and severity of migraine attacks and alleviate associated symptoms such as headaches, nausea and sensitivity to light and sound. However, the current state of the evidence for migraine prevention is poor, making it difficult for those affected and clinicians to make decisions about which medications to consider. Several drugs are recommended by Health Technology Assessment (HTA) agencies within the UK: the National Institute for Health and Care Excellence (NICE) in England and the Scottish Intercollegiate Guidelines Network (SIGN) in Scotland. These include various oral medications used to treat chronic migraine such as Topiramate, Propranolol, Tricyclic antidepressants [9, 13]. The treatment pathway for people with chronic migraine is typically that they have tried at least three of the older, cheap, oral medications before they are able to access Onabotulinum toxin A injections (BTA). Since 2020, calcitonin gene-related peptide (CGRP) monoclonal antibodies (MAbs), such as Erenumab, Fremanezumab, and Galcanezumab have become available and they are usually given as monthly injections [14–17]. These treatment options are more expensive than the earlier generation of oral prophylactic medications. In people with chronic migraine, they are currently reserved for people who have not benefitted from BTA treatment. The availability of these diverse medicines means that there are more choices for healthcare professionals, policymakers and of course, the patients for managing and preventing chronic migraine. Chronic migraine was introduced as a concept in 2007, so many of the oral drugs in earlier studies, have not been trialled under the definition of ‘chronic migraine’. Hence, the current evidence base for the use of oral medications in chronic migraine comes almost exclusively from data extrapolated from trials on episodic migraine.

Evidence regarding the cost-effectiveness of these different pharmacological drugs is also lacking. There are several economic evaluations comparing single prophylactic drugs against another drug or a placebo [13, 18–22]; however, given the range of available treatments, there is an absence of comparing more than one drug. Thus, in this study, based on available evidence we present a more comprehensive economic analysis comparing various prophylactic drugs for chronic migraine in the adult population.

## Methods

The study is reported as per Consolidated Health Economic Evaluation Reporting Standards (CHEERS) 2022 Statement [23]. We have included those drugs included in our network meta-analysis of randomised controlled trials of prophylactic drugs for chronic migraine (manuscript submitted for publication) [24].

### Model structure and assumptions

**Fig. 1:**
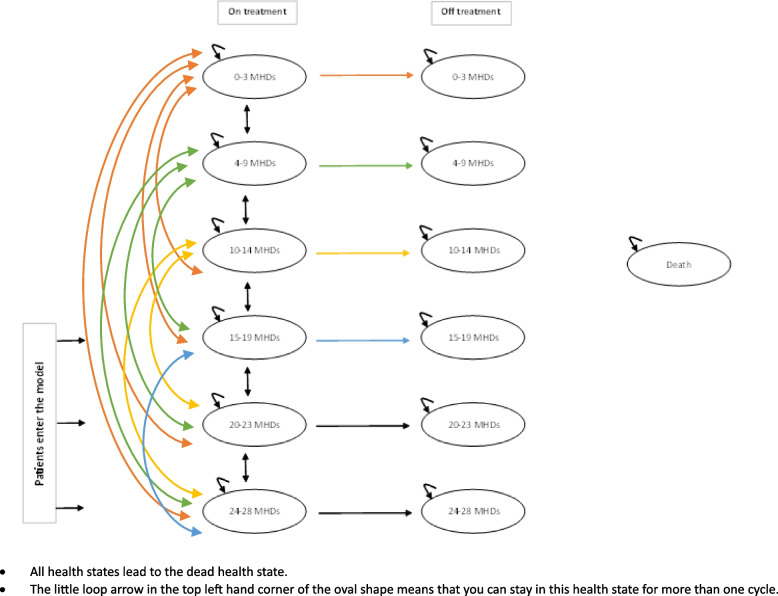
Economic model structure

We built a Markov state-transition model to illustrate the progression of chronic migraine as measured by the number of monthly headache days (MHDs). The model was developed based on a systematic review of economic evaluations of pharmacological drugs for adults with chronic migraine, literature and input from our project team [9, 18, 25]. We created two parallel models for on-treatment and off-treatment scenarios, with MHDs as health states and an additional health state for all-cause mortality. The cycle length for the model was 12 weeks (Fig. 1).

The model starts by assigning a hypothetical cohort of 1,000 people with chronic migraine into one of three chronic migraine health states based on the PREEMPT trial as it is one of the largest chronic migraine trials: 15–19 MHDs – 530 patients; 20–23 MHDs – 280 patients; and 24–28 MHDs—190 patients [26, 27]. We compared the following prophylactic medications in the base-case analysis: i) Onabotulinum toxin A (BTA), ii) Eptinezumab 100mg, iii) Eptinezumab 300mg, iv) Fremanezumab (monthly dose), v) Fremanezumab (quarterly dose), vi) Galcanezumab (120mg), vii) Topiramate (100mg); and viii) placebo. We also compared Erenumab (70mg and 140mg) with data from monthly migraine days in a sensitivity analysis.

### Model inputs

#### Transition probabilities

To estimate transition probabilities: firstly, we digitised the transition probabilities from a paper by Batty et al. [18] which showed a visual representation of the PREEMPT trial [26, 27] transition probabilities for the placebo group. Secondly, for all other pharmacological medications, we derived post-treatment distributions of MHDs for each health state, based on differences in the mean of number of headache days from the network meta-analysis (NMA) conducted as part of our overall chronic migraine project [28]. In other words, for each of the six health states in the ‘on treatment’ arm (0–3, 4–9, 10–14, 15–19, 20–23, 24–28 MHDs) we converted these health states into more granular data. Instead of a cluster of days, a single-day band was used, e.g., 1 headache day per month, 2 headache days per month, and so on. Using this information, we then calculated the transitions (improvement in health, impairment in health and remaining in the same health) required from each headache day per month to either a better or worse health state. For example, a patient having 16 headache days per month (belonging to 15–19 MHDs health state), they would need to reduce their headaches by 2.5 days in a month in order to move to a better health state (10–14 MHDs) or their headaches would need to increase by 3.5 days in order to move into worse health state (20–23 MHDs). Using this information, for each prophylactic medication we worked out the probabilities for a person to move to a better, or to a worse, or to remain in each health state, by calculating weighted probabilities from the distribution. Thirdly, we then applied a discontinuation rate of 10% for BTA and 20% for all other medications based on input from project clinicians which reflected real-life clinical practice. Finally, we multiplied the transition probabilities for the placebo group with the transition probabilities of each pharmacological treatment to obtain transition probabilities for each individual prophylactic drug (see online Supplementary file).

### Health-related quality of life (HRQoL)

The utility values for each of the MHD health states were based on the EQ-5D-5L questionnaire responses from a randomised trial for educational and supportive self-management intervention for people with chronic headaches (CHESS) [29]. The EQ-5D-5L questionnaire includes five questions addressing mobility, self-care, usual activities, pain/discomfort and anxiety/depression, with each dimension assessed at five levels: from no to extreme problems [30]. The EQ-5D-5L responses were converted into health state utilities based on values mapped onto the EQ-5D-3L descriptive system using the the Hernandez-Alava crosswalk algorithm [31]. We assumed that HRQoL was the same for all drugs but varied by MHD health states that the participant was in (see Table 1 for details).

**Table 1:** Utility values used in the base-case analysis

Health states	Mean	SE
0–3 MHD on-treatment	0.7573	0.1662
4–9 MHD on-treatment	0.6449	0.2817
10–14 MHD on-treatment	0.6764	0.2458
15–19 MHD on-treatment	0.6420	0.2543
20–23 MHD on-treatment	0.5916	0.2549
24–28 MHD on-treatment	0.5040	0.2835
0–3 MHD off-treatment	0.7573	0.1662
4–9 MHD off-treatment	0.6449	0.2817
10–14 MHD off-treatment	0.6764	0.2458
15–19 MHD off-treatment	0.6420	0.2543
20–23 MHD off-treatment	0.5916	0.2549
24–28 MHD off-treatment	0.5040	0.2835

### Resource Utilisation and Costs

We obtained drug costs from the British National Formulary [32] and computed them for three-month cycles. Topiramate was the only orally administered drug. All the other medications (except BTA and Eptinezumab), we assumed that the first injection/infusion would be administered by a nurse (30-min) and who would also train the patient on self-administration. We assumed that 10% of patients would not be able to self-administer and accounted for this in each subsequent cycle [33, 34]. For BTA and Eptinezumab, these drugs are administered only in hospitals/clinics (we assumed these would be 15-min appointments with a nurse). The hourly cost of the nurse's time was obtained from the Unit Costs of Health and Social Care 2021 [35]. Costs were adjusted to the 2021/22 price year and any costs outside this period were inflated using the NHS cost inflation index [35] (see Table 2).

**Table 2:** Resource use and unit costs

Resource use item	Unit cost	Source
***Prophylactic drugs (3 monthly cycle) – 2022 prices***		
BTA^c^	£276.40	https://bnf.nice.org.uk/ [32]
Eptinezumab^c^ 100mg	£1,350.00	
Eptinezumab^c^ 300mg	£4,050.00	
Fremanezumab—monthly	£1,350.00	
Fremanezumab—quarterly	£1,350.00	
Galcanezumab	£1,350.00^a^	
Topiramate	£5.10	
***Staff time in 2021/2022 prices***		
Nurse (hourly cost)	£42.00	Unit Costs of Health and Social Care, 2021 [35]
Specialist consultant – neurologist (hourly cost)	£122.00^b^	Latest tariff did not include costs for neurology outpatient therefore assumed to be a Follow Up Attendance—Single Professional (WF01A) for a Neurology outpatient visits (code 400) [36]
***Other resource items in 2021/2022 prices***		
GP visit	£39.23	Unit Costs of Health and Social Care, 2021 [35]
A&E visit	£165.00	A&E worksheet. 'VB08Z', Emergency Medicine, Category 2 Investigation with Category 1 Treatment [37]
Hospital admission	£618.00	Non-elective tariff for code AA31E (Headache, Migraine or Cerebrospinal Fluid Leak, with CC Score 0–6) in worksheet “1 APC & OPROC” HRG code: AA31E [37]
Triptan usage	£3.99	The cost of triptans per attack was based on the weighted average of triptan costs in the UK, taken from NHS Prescriptions Cost Analysis [18, 25]

^a^The cost of maintenance dose in each subsequent cycle

^b^Uprated to 2021/2022 prices

^c^Drugs administered in hospital

Additionally, we allocated a cost of care to each health state for each 12-week cycle, regardless of the prophylactic medication. This cost included visits to GPs, Accident and Emergency (A&E), hospital admissions, and triptan use. The usage frequency of these resources was obtained from the International Burden of Migraine study (IBMS) for UK patients and in line with published NICE guidance [25, 38–40].

We also consulted the NICE guidance [25, 39, 40] for the different prophylactic medications and included any additional visits from neurology consultants and nurses (see Table 2).

### All-cause mortality

The model used age-specific mortality rates obtained from the Office for National Statistics (ONS) in the UK [41]. The rates were based on general population lifetime tables and averaged for males and females. Mortality rates increase as the cohort ages over the model's time horizon.

### Base-case and sensitivity analysis

The Markov model adopted a UK NHS and Personal Social Service (PSS) perspective to analyse the costs and quality-adjusted life years (QALYs) of various prophylactic drugs for chronic migraine. The analysis used a two-year time horizon and a starting age of 30 years for the patient cohort. The costs were measured in 2021/2022 prices and health outcomes in QALYs. The cost-effectiveness analysis was measured in terms of an incremental cost per QALY gained (ICER), with a discount rate of 3.5% applied to both costs and outcomes.

To account for uncertainty in model parameters and sampling variability, we did a probabilistic sensitivity analysis (PSA) using Monte Carlo simulations with 1,000 iterations for all model inputs, except for drug costs which were fixed values. A gamma distribution was applied for costs, and a beta distribution was used for utility values. A cost-effectiveness acceptability frontier (CEAF) was used to summarise the uncertainty for the different medications jointly, by indicating which medication is preferred at different threshold values for cost-effectiveness. The cost per QALY threshold by NICE for England and Wales is between £20-30k.

### Scenario and sensitivity analyses

We did scenario and sensitivity analyses by altering base-case inputs into the model:

1. Changing time horizon – from a 2-year time horizon to a 5-year and a life-time horizon.
2. Utility inputs – using van-Hout crosswalk algorithm [42] instead of the Hernandez-Alava crosswalk algorithm [31].
3. Monthly Migraine Days (MMDs) – using MMDs as the outcome measure instead of MHDs, allowed us to include Erenumab—70mg and 140mg in the analysis. Additionally, we utilised utility values based on MMDs from the Lipton et al. study [43].
4. Reducing drug costs for CGRP MAbs– confidential discounts are agreed via the Patient Access Scheme between the NHS and manufacturers, but their actual value is not available. We reduced the costs of the following drugs by 50%: Eptinezumab 100mg and 300mg, Fremanezumab monthly and quarterly, and Galcanezumab.

## Results

### Base-case analysis – comparing each medication separately to placebo

The deterministic discounted results showed that Topiramate dominated placebo as it was cheaper (£104 less expensive) and more effective (0.0464 more QALYs). The other medications were more expensive than placebo, however, they generated additional QALYs when compared to placebo. BTA was more cost-effective than placebo at the £30k threshold with an ICER of £25,238 per QALY gained. The other five medications (Fremanzumab monthly, Fremanzumab quarterly, Eptinezumab 100mg, Eptinezumab 300mg and Galcanuzmab) when compared with placebo had ICERs which would not be considered cost-effective if using a £20-30k ($50k or $100) per QALY threshold used by NICE in the UK (threshold values used in the USA [44]). Probabilistic results where similar to deterministic results (see Table 3).

**Table 3:** Base-case cost-effectiveness results comparing each medication separately

	**Costs (£)**	**QALYs**	**Incremental costs (£)**	**Incremental QALYs**	**ICER: cost per QALY gained (£)**
**Deterministic results—discounted**					
Placebo	£1,729	1.3531	-	-	-
Topiramate	£1,624	1.3995	-£104	0.0464	Dominated
Placebo	£1,729	1.3531	-	-	-
BTA	£3,654	1.4294	£1,925	0.0763	£25,238
Placebo	£1,729	1.3531	-	-	-
Fremanezumab (monthly)	£10,155	1.4307	£8,427	0.0776	£108,604
Placebo	£1,729	1.3531	-	-	-
Fremanezumab (quarterly)	£10,193	1.4224	£8,465	0.0693	£122,126
Placebo	£1,729	1.3531	-	-	-
Eptinezumab 100	£10,216	1.4239	£8,487	0.0708	£119,796
Placebo	£1,729	1.3531	-	-	-
Galcanezumab	£10,640	1.4229	£8,912	0.0698	£127,649
Placebo	£1,729	1.3531	-	-	-
Eptinezumab 300	£27,401	1.4403	£25,672	0.0873	£294,151
**Probabilistic results—discounted**					
Placebo	£1,728	1.3460	-	-	-
Topiramate	£1,624	1.4045	-£104	0.0584	Dominated
Placebo	£1,728	1.3460	-	-	-
BTA	£3,654	1.4270	£1,926	0.0810	£23,775
Placebo	£1,728	1.3460	-	-	-
Fremanezumab (monthly)	£10,161	1.4350	£8,433	0.0890	£94,748
Placebo	£1,728	1.3460	-	-	-
Fremanezumab (quarterly)	£10,196	1.4273	£8,467	0.0812	£104,251
Placebo	£1,728	1.3460	-	-	-
Eptinezumab 100	£10,221	1.4199	£8,492	0.0739	£114,894
Placebo	£1,728	1.3460	-	-	-
Galcanezumab	£10,646	1.4161	£8,917	0.0701	£127,279
Placebo	£1,728	1.3460	-	-	-
Eptinezumab 300	£27,411	1.4365	£25,683	0.0904	£284,030

### Base-case analysis – comparing all medications together

**Fig. 2:**
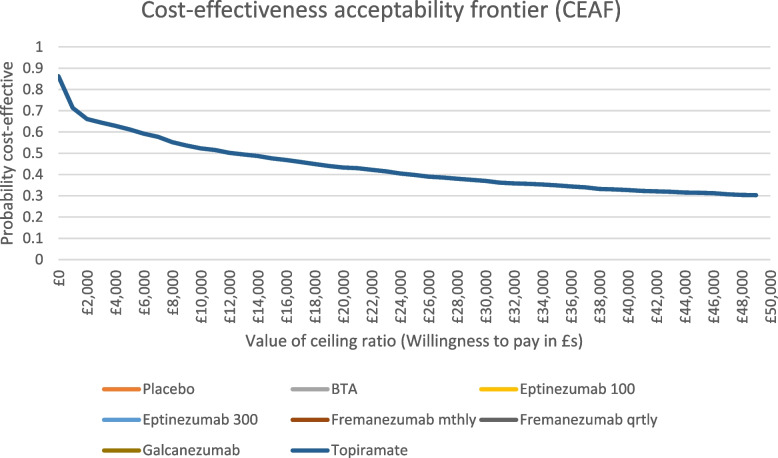
Base-case cost-effectiveness acceptability frontier

Table 4 shows the discounted deterministic results when comparing all medications ranked by the least costly option. Topiramate was the least costly option and had slightly more QALYs than the placebo, whereas Eptinezumab 300mg was the more costly option and generated the most QALYs. Options placebo (dominated by Topiramate), Fremanezumab quarterly, Eptinezumab 100mg and Galcanezumab (all dominated by Fremanezumab monthly) were all eliminated as they were dominated by other medications. We then compared Topiramate, BTA, Fremanezumab monthly and Eptinezumab 300mg. Fremanezumab monthly was extendedly dominated (where any interventions that have an ICER which is greater than that of a more effective intervention is ruled out) by a linear combination of BTA and Eptinezumab 300mg and was therefore eliminated. The ICER between BTA and Topiramate was £68,000 per QALY gained and the ICER between Eptinezumab 300mg and BTA was not within plausible cost-effectiveness thresholds. The probabilistic results were similar to the deterministic results. The CEAF shows that Topiramate is the most cost-effective medication for any amount the decision maker is willing-to-pay per QALY (see Fig. 2).

**Table 4:** Base-case cost-effectiveness results comparing all medications

	**Costs (£)**	**QALYs**	**Incremental costs (£)**	**Incremental QALYs**	**ICER: cost per QALY gained (£)**	**Comparison**
**Deterministic results—discounted**						
Topiramate	£1,625	1.3995	-	-	-	
Placebo	£1,729	1.3531	£104	-0.0464	Dominated	Placebo vs. Topiramate
BTA	£3,654	1.4294	£2,029	0.0298	£68,002	BTA vs. Topiramate
Fremanezumab (monthly)	£10,155	1.4403	£6,501	0.0013	Extendedly dominated	Fremanezumab (monthly) vs. BTA
Fremanezumab (quarterly)	£10,193	1.4224	£38	-0.0083	Dominated	Fremanezumab (quarterly vs. monthly)
Eptinezumab 100	£10,216	1.4239	£22	-0.0067	Dominated	Eptinezumab 100 vs Fremanezumab (monthly)
Galcanezumab	£10,640	1.4229	£485	-0.0078	Dominated	Galcanezumab vs. Fremanezumab (monthly)
Eptinezumab 300	£27,401	1.4403	£17,246	0.0097	£2,160,037	Eptinezumab 300 vs BTA
**Probabilistic results—discounted**						
Topiramate	£1,624	1.4045	-	-	-	
Placebo	£1,728	1.3460	£104	-0.0584	Dominated	Placebo vs. Topiramate
BTA	£3,654	1.4270	£2,030	0.0226	£89,939	BTA vs. Topiramate
Fremanezumab (monthly)	£10,161	1.4350	£6,507	0.0080	Extendedly dominated	Fremanezumab (monthly) vs. BTA
Fremanezumab (quarterly)	£10,196	1.4273	£34	-0.0078	Dominated	Fremanezumab (quarterly vs. monthly)
Eptinezumab 100	£10,221	1.4199	£59	-0.0151	Dominated	Eptinezumab 100 vs Fremanezumab (monthly)
Galcanezumab	£10,646	1.4161	£485	-0.0189	Dominated	Galcanezumab vs. Fremanezumab (monthly)
Eptinezumab 300	£27,411	1.4365	£17,250	0.0014	£2,524,429	Eptinezumab 300 vs BTA

Extendedly dominated is where any interventions that have an ICER which is greater than that of a more effective intervention is ruled out

### Sensitivity analysis

Table 5 shows the results for the discounted probabilistic sensitivity analysis when comparing all medications together (the discounted deterministic results were similar and have not been presented here). For all the different scenarios, and in line with the base-case results, Topiramate was the least costly option and had slightly more QALYs than placebo; whereas Eptinezumab 300mg was the more costly option. For all scenarios, when removing the dominated options, BTA was more cost-effective than Topiramate; however, the cost per QALY gained was not within plausible thresholds unless a lifetime horizon was used. After removing the dominated options, when BTA was compared with either Fremanezumab monthly or Eptinezumab 300mg, the ICERs were not within plausible cost-effectiveness threshold ranges.

**Table 5:** Sensitivity analysis results comparing all medications

	**Costs (£)**	**QALYs**	**Incremental costs (£)**	**Incremental QALYs**	**ICER: cost per QALY gained (£)**	**Comparison**
**a) 5-year time horizon**						
**Probabilistic results – discounted**						
Topiramate	£3,159	3.1717	-	-	-	-
Placebo	£3,491	3.0348	£333	-0.1369	Dominated	Placebo vs. Topiramate
BTA	£6,383	3.2497	£3,224	0.0779	£41,366	BTA vs. Topiramate
Fremanezumab (monthly)	£16,039	3.2483	£9,656	-0.0014	Dominated	Fremanezumab (monthly) vs. BTA
Fremanezumab (quarterly)	£16,120	3.2283	£9,737	-0.0214	Dominated	Fremanezumab (quarterly) vs BTA
Eptinezumab 100	£16,145	3.2163	£9,762	-0.0334	Dominated	Eptinezumab 100 vs. BTA
Galcanezumab	£16,577	3.2071	£10,194	-0.0425	Dominated	Galcanezumab vs. BTA
Eptinezumab 300	£42,184	3.2573	£35,801	0.0076	£4,707,286	Eptinezumab 300 vs. BTA
b) **Lifetime horizon**						
**Probabilistic results—discounted**						
Topiramate	£13,351	15.7628	-	-	-	-
Placebo	£15,138	15.1467	£1,787	-0.6161	Dominated	Placebo vs. Topiramate
BTA	£16,381	16.2613	£3,030	0.4985	£6,077	BTA vs. Topiramate
Fremanezumab (monthly)	£27,469	16.1774	£11,088	-0.0840	Dominated	Fremanezumab (monthly) vs. BTA
Eptinezumab 100	£27,846	16.1319	£11,465	-0.1294	Dominated	Fremanezumab (quarterly) vs BTA
Fremanezumab (quarterly)	£27,840	16.0931	£11,459	-0.1682	Dominated	Eptinezumab 100 vs. BTA
Galcanezumab	£28,194	16.1418	£11,813	-0.1195	Dominated	Galcanezumab vs. BTA
Eptinezumab 300	£57,609	16.3428	£41,228	0.0815	£505,711	Eptinezumab 300 vs. BTA
c) **Utility inputs—van-Hout crosswalk algorithm**						
**Probabilistic results—discounted**						
Topiramate	£1,627	1.4063	-	-	-	-
Placebo	£1,723	1.3807	96	-0.0256	Dominated	Placebo vs. Topiramate
BTA	£3,656	1.4475	£2,029	0.0412	£49,265	BTA vs. Topiramate
Fremanezumab (monthly)	£10,161	1.4608	£6,505	0.0133	Extendedly dominated	Fremanezumab (monthly) vs. BTA
Fremanezumab (quarterly)	£10,193	1.4532	£32	-0.0076	Dominated	Fremanezumab (quarterly) vs Fremanezumab (monthly)
Eptinezumab 100	£10,221	1.4346	£60	-0.0262	Dominated	Eptinezumab 100 vs. Fremanezumab (monthly)
Galcanezumab	£10,650	1.4436	£489	-0.0172	Dominated	Galcanezumab vs. Fremanezumab (monthly)
Eptinezumab 300	£27,411	1.4512	£17,250	-0.0096	£6,353,726	Eptinezumab 300 vs. BTA
d) **Using MMDs instead of MHDs**						
**Probabilistic results—discounted**						
Topiramate	£1,585	1.3220	-	-	-	-
Placebo	£1,731	1.2245	£146	-0.0975	Dominated	Placebo vs. Topiramate
BTA	£3,645	1.3566	£2,060	0.0346	£59,596	BTA vs. Topiramate
Erenumab 70	£8,944	1.3754	£5,299	0.0188	Extendedly dominated	Erenumab 70 vs BTA
Erenumab 140	£8,949	1.3749	£5	-0.0005	Dominated	Erenumab 140 vs Erenumab 70
Fremanezumab (monthly)	£10,072	1.3916	£1,128	0.0162	£183,732	Fremanezumab (monthly) vs. BTA
Fremanezumab (quarterly)	£10,140	1.3644	£68	-0.0272	Dominated	Fremanezumab (quarterly) vs Fremanezumab (monthly)
Eptinezumab 100	£10,188	1.3584	£116	-0.0332	Dominated	Eptinezumab 100 vs. Fremanezumab (monthly)
Galcanezumab	£10,610	1.3584	£538	-0.0332	Dominated	Galcanezumab vs. Fremanezumab (monthly)
Eptinezumab 300	£27,377	1.3850	£17,305	-0.0065	Dominated	Eptinezumab 300 vs. Fremanezumab (monthly)
e) **Reducing costs of MAbs by 50%**						
**Probabilistic results—discounted**						
Topiramate	£1,625	1.4078	-	-	-	-
Placebo	£1,729	1.3415	£105	-0.0663	Dominated	Placebo vs. Topiramate
BTA	£3,653	1.4218	£2,028	0.0140	£144,881	BTA vs. Topiramate
Fremanezumab (monthly)	£5,835	1.4395	£2,182	0.0177	£123,111	Fremanezumab (monthly) vs. BTA
Fremanezumab (quarterly)	£5,869	1.4321	£34	-0.0074	Dominated	Fremanezumab (quarterly) vs Fremanezumab (monthly)
Eptinezumab 100	£5,896	1.4210	£61	-0.0185	Dominated	Eptinezumab 100 vs. Fremanezumab (monthly)
Galcanezumab	£6,097	1.4272	£261	-0.0123	Dominated	Galcanezumab vs. Fremanezumab (monthly)
Eptinezumab 300	£14,455	1.4358	£8,620	-0.0037	Dominated	Eptinezumab 300 vs. Fremanezumab (monthly)

Extendedly dominated is where any interventions that have an ICER which is greater than that of a more effective intervention is ruled out

## Discussion

In this economic evaluation we aimed to determine the cost-effectiveness of different pharmacological drugs for managing chronic migraine. With numerous drugs available for chronic migraine management in the UK, it can be challenging to determine the most cost-effective option, while ensuring that the limited resources and finite budget meets the needs of chronic migraine patients. Our 2022 review of existing economic analyses for chronic migraine prophylactic medications revealed a lack of comprehensive evaluations that compared more than three medications against each other [9]. In the absence of such evidence, this study provides more comprehensive insights into managing a common neurological disorder. It also has important implications for policymakers in helping them making informed decisions and allocating scarce resources for chronic migraine management. It can help optimise patient access to effective treatments while ensuring efficient utilisation of healthcare resources. This approach has the potential to enhance the overall quality of care provided to individuals suffering from chronic migraine, leading to better access to treatments, improved quality of life and better allocation of limited healthcare resources.

For the base-case analysis, the deterministic results showed when comparing each of the medications seperately against placebo, Topiramate dominated placebo. The other drugs when compared separately, were more expensive than placebo, however, they generated more QALYs. In terms of the cost per QALY gained, BTA was more cost-effective than placebo at the £30k threshold with an ICER of £25,328 per QALY gained. The deterministic results when comparing all medications together, Topiramate was the cheapest, but generated the fewest QALYs (with the exception of placebo). On the other hand, Eptinezumab 300mg was the most expensive option and produced the most QALYs. The ICER for BTA vs Topiramate was estimated to be £68,000 per QALY gained, while the ICER for Eptinezumab 300mg vs BTA was not within plausible cost-effectiveness thresholds. The CEAF revealed that when comparing all medications, Topiramate was most likely to be the cost-effective medication for any amount the decision-maker is willing-to-pay per QALY. NICE typically uses a threshold range of £20-£30k per QALY gained as a reference range, but this range can be higher or lower depending on the circumstances. Base-case probabilistic results were consistent with the base-case deterministic results. Sensitivity and scenario analyses were conducted, primarily using MHDs as an outcome measure, and the results were mostly consistent with the base-case findings. The only important exception was that when using MMDs as an outcome measure, Fremanezumab monthly generated more QALYs than Eptinezumab 300mg.

Our results are in line with previous studies. Batty et al. (2013) concluded the use of BTA for chronic migraine resulted in an increase in costs of £1,367 and an improvement in QALYs of 0.1 compared to placebo, resulting in an ICER of £15,028. Specifically, treatment with BTA was associated with a reduction in headache days by approximately 38 days per year, at a cost of £18 per headache day avoided [18]. A 2018 study found that with an annual drug price of US$6,900 (£5,604 in 2017 prices) for Erenumab in 2017 prices, treatment with Erenumab compared to no preventive treatment is dominant from a societal perspective, meaning it is both cheaper and more effective for chronic migraine patients. When indirect costs were excluded, the ICERs were considered to be cost-effective for chronic migraine participants: comparing Erenumab to no preventive treatment the ICER was (US$23,079; £18,746 in 2017 prices) and when comparing Erenumab with BTA, although the ICER (US$65,720; £53,380 in 2017 prices) was considered cost-effective, it is not within current UK cost-effectiveness thresholds [22].

The 2022 systematic review on this topic by our team concluded that BTA is cost-effective when compared to a placebo, with an ICER ranging between £15,028 and £16,598 [9]. For individuals who did not respond to previous preventive treatments, Erenumab was shown to be a cost-effective alternative to placebo. However, when comparing Erenumab to BTA, the ICERs ranged from £59,712 to £182,128, exceeding the most commonly accepted willingness-to-pay (WTP) thresholds [9]. Under widely accepted WTP thresholds, all CGRP MAbs, including Erenumab, Galcanezumab, and Fremanezumab, were deemed cost-effective for the chronic migraine population who have failed BTA [9].

### Strengths of the study

To our knowledge, this is the first study that encompasses five drugs (seven different dosing regimens) plus placebo for managing chronic migraine providing valuable insights into cost-effectiveness. The study addressed a gap in the literature by comparing multiple medications against each other, offering a more comprehensive analysis of available options. The study features sensitivity analyses, which enable a wide range of changes in the parameters of interest to be examined and their potential impact on the base-case results to be investigated. Sensitivity and scenario analyses confirmed the robustness of the findings as the probabilistic results were consistent with the base-case deterministic results.

### Study limitations

Due to the lack of readily available evidence in the literature we had to employ some additional assumptions, some of which may not be true in current practice. Firstly, one assumption we used was when someone enters the ‘off-treatment’ health state, they cannot return to an ‘on-treatment’ health state. For example, we know that a participant can come off a prophylactic medication if their migraines are better, or if they cannot tolerate a medication; however, their migraine may return sometime later, and they may be prescribed another medication for their migraine.

Secondly, we assumed that the treatment effects were based on mean health differences from our NMA, where we assumed these effects would be uniformly distributed across all health states, regardless of the severity of the condition at the start. However, it is likely that there will be heterogeneity in the distribution of effects. Furthermore, in our NMA we have not included evidence on other oral medications (such as Amitriptyline, Candesartan Propranolol). We only included trials with at least 100 participants per arm meaning it was possible we excluded some smaller studies of other oral medications. However, on re-checking the excluded studies list there were no trials excluded from the NMA on the basis of size alone [28].

Thirdly, the small differences in QALYs between some of the medications namely Fremanezumab and BTA meant that they produced very large ICERs. Even quite small changes in the QALY estimate might substantially change the apparent cost-effectiveness. Fourthly, we used utility data based on MHDs based on the CHESS trial. There was limited data in the literature on utility values for MMDs; the majority of utility values for MMDs were based on data for episodic migraine [19, 22, 43] Also, there were no studies that mapped EQ-5D or SF-6D data to generate utility values for the specific headache day health states we have used in our model.

Fifthly, we only considered a NHS and PSS perspective. If we were to take a broader societal perspective, incorporating indirect costs such as productivity losses, the resulting ICERs may have been different. Finally, we excluded adverse events from the model, based on evidence from our systematic review on adverse events, we found that serious adverse events were not related to the medication itself and therefore were assumed to not influence health care resource usage [22, 28].

## Conclusion

Among the different prophylactic medications for managing chronic migraine included in this study, it seems that Topiramate was the cheapest, however, it is not the most effective in terms of gained QALYs in comparison with other medications. On the other hand, Eptinezumab 300mg was more costly, however, it accrued the most QALYs. When comparing all medications, only Topiramate was within typical cost-effectiveness threshold ranges. Further research is needed, ideally an economic evaluation alongside a randomised trial, to compare these newer, expensive CGRP MAbs with the cheaper oral medications.

### Supplementary Information

**Additional file 1: Table A**. Deterministic transition probabilities used in the base-case analysis.

## Data Availability

The datasets used and/or used during the current study are available from the data sharing committee on reasonable request.
