# Supplementary material for: Competing treatments for migraine: a headache for decision-makers
Source: J Headache Pain. 2023 Dec 5;24(1):162. doi: 10.1186/s10194-023-01686-y (PMC10696771; doi:10.1186/s10194-023-01686-y)
Supplement: Supplementary file 1 — Additional file 1: Table A. Deterministic transition probabilities used in the base-case analysis. [file 10194_2023_1686_MOESM1_ESM.docx]

Table A: Deterministic transition probabilities used in the base-case analysis

| **Transitions** | **Placebo** | **BTA** | **Eptinezumab 100mg** | **Eptinezumab 300mg** | **Fremanezumab (monthly)** | **Fremanezumab (quarterly)** | **Galcanezumab** | **Topiramate** |
| --- | --- | --- | --- | --- | --- | --- | --- | --- |
| 0-3 MHD to 0-3 MHD | 0.56200 | 0.68563 | 0.67243 | 0.69976 | 0.68342 | 0.66996 | 0.67054 | 0.62965 |
| 0-3 MHD to 4-9 MHD | 0.28100 | 0.16337 | 0.16182 | 0.14079 | 0.15336 | 0.16373 | 0.16336 | 0.19681 |
| 0-3 MHD to 10-14 MHD | 0.05400 | 0.03862 | 0.03823 | 0.03368 | 0.03640 | 0.03863 | 0.03847 | 0.04386 |
| 0-3 MHD to 15-19 MHD | 0.01500 | 0.01660 | 0.01614 | 0.01934 | 0.01743 | 0.01586 | 0.01604 | 0.01363 |
| 0-3 MHD to 20-23 MHD | 0.03400 | 0.02601 | 0.02585 | 0.02090 | 0.02386 | 0.02629 | 0.02605 | 0.03053 |
| 0-3 MHD to 24-28 MHD | 0.00000 | 0.00000 | 0.00000 | 0.00000 | 0.00000 | 0.00000 | 0.00000 | 0.00000 |
| 0-3 MHD to 0-3 Off TX | 0.05400 | 0.06977 | 0.08553 | 0.08553 | 0.08553 | 0.08553 | 0.08553 | 0.08553 |
| 4-9 MHD to 0-3 MHD | 0.17200 | 0.40152 | 0.39196 | 0.43971 | 0.41116 | 0.38764 | 0.38866 | 0.31857 |
| 4-9 MHD to 4-9 MHD | 0.49100 | 0.33880 | 0.33422 | 0.31423 | 0.32618 | 0.33606 | 0.33601 | 0.37326 |
| 4-9 MHD to 10-14 MHD | 0.23800 | 0.15606 | 0.15482 | 0.13033 | 0.14497 | 0.15701 | 0.15615 | 0.18513 |
| 4-9 MHD to 15-19 MHD | 0.02800 | 0.02325 | 0.02281 | 0.02362 | 0.02314 | 0.02274 | 0.02283 | 0.02300 |
| 4-9 MHD to 20-23 MHD | 0.02800 | 0.02483 | 0.02460 | 0.02122 | 0.02324 | 0.02490 | 0.02471 | 0.02738 |
| 4-9 MHD to 24-28 MHD | 0.00600 | 0.00249 | 0.00249 | 0.00179 | 0.00221 | 0.00255 | 0.00254 | 0.00355 |
| 4-9 MHD to 4-9 Off TX | 0.03700 | 0.05305 | 0.06910 | 0.06910 | 0.06910 | 0.06910 | 0.06910 | 0.06911 |
| 10-14 MHD to 0-3 MHD | 0.08400 | 0.21276 | 0.20761 | 0.23435 | 0.21836 | 0.20519 | 0.20576 | 0.16652 |
| 10-14 MHD to 4-9 MHD | 0.27500 | 0.26762 | 0.26233 | 0.27559 | 0.26766 | 0.26118 | 0.26200 | 0.25391 |
| 10-14 MHD to 10-14 MHD | 0.34300 | 0.27936 | 0.27567 | 0.25747 | 0.26835 | 0.27729 | 0.27666 | 0.29818 |
| 10-14 MHD to 15-19 MHD | 0.18700 | 0.12471 | 0.12346 | 0.10850 | 0.11744 | 0.12480 | 0.12437 | 0.14399 |
| 10-14 MHD to 20-23 MHD | 0.04700 | 0.04675 | 0.04622 | 0.04159 | 0.04436 | 0.04662 | 0.04634 | 0.04928 |
| 10-14 MHD to 24-28 MHD | 0.01900 | 0.00789 | 0.00789 | 0.00567 | 0.00699 | 0.00809 | 0.00804 | 0.01126 |
| 10-14 MHD to 10-14 Off TX | 0.04500 | 0.06092 | 0.07683 | 0.07683 | 0.07683 | 0.07683 | 0.07683 | 0.07686 |
| 15-19 MHD to 0-3 MHD | 0.02100 | 0.08076 | 0.07868 | 0.09103 | 0.08364 | 0.07756 | 0.07782 | 0.05973 |
| 15-19 MHD to 4-9 MHD | 0.12700 | 0.16688 | 0.16293 | 0.18265 | 0.17086 | 0.16118 | 0.16204 | 0.14218 |
| 15-19 MHD to 10-14 MHD | 0.27500 | 0.28304 | 0.27802 | 0.28198 | 0.27961 | 0.27766 | 0.27780 | 0.27311 |
| 15-19 MHD to 15-19 MHD | 0.30900 | 0.21684 | 0.21418 | 0.19665 | 0.20713 | 0.21577 | 0.21541 | 0.24132 |
| 15-19 MHD to 20-23 MHD | 0.12700 | 0.13237 | 0.13077 | 0.11949 | 0.12624 | 0.13174 | 0.13100 | 0.13715 |
| 15-19 MHD to 24-28 MHD | 0.06200 | 0.02575 | 0.02573 | 0.01850 | 0.02283 | 0.02639 | 0.02623 | 0.03673 |
| 15-19 MHD to 15-19 Off TX | 0.07900 | 0.09435 | 0.10970 | 0.10970 | 0.10970 | 0.10970 | 0.10970 | 0.10978 |
| 20-23 MHD to 0-3 MHD | 0.00000 | 0.03029 | 0.02942 | 0.03564 | 0.03192 | 0.02885 | 0.02899 | 0.01989 |
| 20-23 MHD to 4-9 MHD | 0.06400 | 0.06680 | 0.06541 | 0.06992 | 0.06723 | 0.06502 | 0.06526 | 0.06167 |
| 20-23 MHD to 10-14 MHD | 0.09200 | 0.17809 | 0.17350 | 0.20102 | 0.18456 | 0.17104 | 0.17200 | 0.13944 |
| 20-23 MHD to 15-19 MHD | 0.32800 | 0.26864 | 0.26370 | 0.27076 | 0.26654 | 0.26312 | 0.26398 | 0.26828 |
| 20-23 MHD to 20-23 MHD | 0.31100 | 0.34413 | 0.33962 | 0.31612 | 0.33018 | 0.34164 | 0.33991 | 0.34896 |
| 20-23 MHD to 24-28 MHD | 0.18700 | 0.07768 | 0.07762 | 0.05580 | 0.06884 | 0.07959 | 0.07912 | 0.11078 |
| 20-23 MHD to 20-23 Off TX | 0.01800 | 0.03437 | 0.05073 | 0.05073 | 0.05073 | 0.05073 | 0.05073 | 0.05098 |
| 24-28 MHD to 0-3 MHD | 0.00000 | 0.00000 | 0.00000 | 0.00000 | 0.00000 | 0.00000 | 0.00000 | 0.00000 |
| 24-28 MHD to 4-9 MHD | 0.00000 | 0.00891 | 0.00860 | 0.01140 | 0.00972 | 0.00835 | 0.00845 | 0.00514 |
| 24-28 MHD to 10-14 MHD | 0.02400 | 0.04885 | 0.04757 | 0.05550 | 0.05075 | 0.04686 | 0.04713 | 0.03775 |
| 24-28 MHD to 15-19 MHD | 0.09200 | 0.08665 | 0.08464 | 0.09417 | 0.08847 | 0.08382 | 0.08443 | 0.07881 |
| 24-28 MHD to 20-23 MHD | 0.13900 | 0.50391 | 0.49180 | 0.55323 | 0.51651 | 0.48619 | 0.48697 | 0.38584 |
| 24-28 MHD to 24-28 MHD | 0.70000 | 0.29077 | 0.29055 | 0.20887 | 0.25771 | 0.29795 | 0.29619 | 0.41468 |
| 24-28 MHD to 24-28 Off TX | 0.04500 | 0.06092 | 0.07683 | 0.07683 | 0.07683 | 0.07683 | 0.07684 | 0.07777 |
| 0-3 Off TX to 0-3 Off TX | 1.00000 | 1.00000 | 1.00000 | 1.00000 | 1.00000 | 1.00000 | 1.00000 | 1.00000 |
| 4-9 Off TX to 4-9 Off TX | 1.00000 | 1.00000 | 1.00000 | 1.00000 | 1.00000 | 1.00000 | 1.00000 | 1.00000 |
| 10-14 Off TX to 10-14 Off TX | 1.00000 | 1.00000 | 1.00000 | 1.00000 | 1.00000 | 1.00000 | 1.00000 | 1.00000 |
| 15-19 Off TX to 15-19 Off TX | 1.00000 | 1.00000 | 1.00000 | 1.00000 | 1.00000 | 1.00000 | 1.00000 | 1.00000 |
| 20-23 Off TX to 20-23 Off TX | 1.00000 | 1.00000 | 1.00000 | 1.00000 | 1.00000 | 1.00000 | 1.00000 | 1.00000 |
| 24-28 Off TX to 24-28 Off TX | 1.00000 | 1.00000 | 1.00000 | 1.00000 | 1.00000 | 1.00000 | 1.00000 | 1.00000 |

MHD = Monthly headache day; Off TX = off treatment
